# Improving the feasibility of deprescribing proton pump inhibitors: GPs’ insights on barriers, facilitators, and strategies

**DOI:** 10.3389/fphar.2024.1468750

**Published:** 2024-09-20

**Authors:** Nuša Japelj, Lea Knez, Davorina Petek, Nejc Horvat

**Affiliations:** ^1^ Department of Social Pharmacy, Faculty of Pharmacy, University of Ljubljana, Ljubljana, Slovenia; ^2^ Department of Pharmacy, University Clinic Golnik, Golnik, Slovenia; ^3^ Department of Family Medicine, Faculty of Medicine, University of Ljubljana, Ljubljana, Slovenia

**Keywords:** deprescriptions, primary healthcare, proton pump inhibitors, inappropriate prescribing, qualitative research

## Abstract

**Introduction:**

The prevalent overprescribing of proton pump inhibitors (PPIs) poses health risks from prolonged use. GPs play a key role in initiating deprescribing PPIs, so understanding their decision-making factors and strategies to improve feasibility is crucial. This study aimed to investigate the perspectives of GPs on deprescribing PPIs with a focus on identifying facilitators, barriers, and strategies to enhance feasibility in clinical settings.

**Methods:**

A qualitative study involving semi-structured interviews was conducted with nine GPs or trainees. The thematic analysis of the interviews was conducted using NVivo R1 (2020).

**Results:**

Four main categories were identified: 1) Inappropriate prescribing of PPIs, 2) Facilitators for deprescribing PPIs, 3) Barriers to deprescribing PPIs, 4) Feasibility of deprescribing PPIs. GPs acknowledged excessive and often inappropriate PPI prescribing, with a lack of deprescribing efforts mainly due to time constraints. Other key barriers included patient reluctance, fear of symptom recurrence, and unawareness of long-term risks. Patient-initiated request is key facilitator for deprescribing PPIs. GPs emphasized the need for collaboration with healthcare professionals, clear guidelines, improved digital support, increased physician availability, and raising awareness among providers and patients to enhance deprescribing feasibility.

**Discussion:**

GPs are calling for a multifaceted approach to improve the feasibility of deprescribing PPIs, involving patient-centered approaches, systemic optimizations, support from other healthcare professionals, and provider-centered strategies to emphasize the importance of deprescribing PPIs.

## 1 Introduction

Proton pump inhibitors (PPIs) rank among the most frequently prescribed groups of medicines, notably with pantoprazole being second most prescribed medicine in Slovenia in 2023 (Kostnapfel and Albreht, 2024). PPIs are widely used for treating upper gastrointestinal tract conditions, such as gastroesophageal reflux disease and esophagitis, eradicating *Helicobacter pylori* infections in combination with antibiotics, and preventing stress ulcers in hospital settings (Farrell et al., 2017).

A notable challenge is the prevalent use of PPIs without valid indications, as observed in nearly 50% of patients in primary healthcare settings (Heidelbaugh et al., 2012). This misuse not only contributes to an increased pill burden, which can lead to nonadherence, medication errors, and more frequent emergency department visits and hospitalizations, but their long-term use is also associated with several adverse effects, including hypomagnesemia, osteoporotic fractures, serious infections, and the malabsorption of essential nutrients (Hayes et al., 2019; Lee and McDonald, 2020). If adverse effects are not recognized, prescribing cascades may occur, where new medicines are prescribed to treat the side effects of PPIs instead of stopping the initial PPI, potentially causing more side effects and complications (Doherty et al., 2022). PPIs are also prone to drug-drug interactions: they can reduce the absorption of other medicines by increasing gastric pH, such as HIV protease inhibitors (Wolfe, 2024) or tyrosine kinase inhibitors (Raoul et al., 2023); affect the metabolism of active substances through their effects on hepatic cytochrome P450 enzymes, potentially leading to serious cardiovascular events in patients treated concurrently with clopidogrel and omeprazole (Muthiah et al., 2021); and cause slower elimination of certain medicines, such as high-dose methotrexate, potentially increasing its toxicity (Wolfe, 2024). On a pharmacodynamic level, recent research has underlined the detrimental effects of PPI usage on the gut microbiome, which may reduce the efficacy of cancer immunotherapy, leading to worse overall survival (Weersma et al., 2020; Hopkins et al., 2022; Chalabi et al., 2020). Therefore, PPIs should be prescribed carefully, at the lowest effective dose and for the shortest recommended duration (Scott et al., 2015). Generally, conditions requiring PPIs call for short-term treatment, often not exceeding a few weeks, and upon symptom resolution, deprescribing PPIs should be considered (Farrell et al., 2017; Targownik et al., 2022). Deprescribing is defined as the planned and supervised process of dose reduction or stopping of medicine that might be causing harm or might no longer be providing benefit, aiming to decrease medication burden and harm while maintaining or enhancing quality of life (Farrell et al., 2017).

Several strategies for deprescribing PPIs have been described, including abrupt discontinuation, gradual dose reduction, or on-demand use (Haastrup et al., 2014; Wilsdon et al., 2017). The implementation of feasible interventions to effectively deprescribe PPIs in clinical settings remains a significant challenge and has not yet been routinely established (Raghunath et al., 2005; Gendre et al., 2022). Considering that GPs handle almost all PPI prescriptions (Gendre et al., 2022), the facilitators and barriers they perceive in embracing deprescribing PPIs are essential. This study aimed to investigate the perspectives of GPs on deprescribing PPIs with a focus on identifying facilitators, barriers, and strategies to enhance feasibility in clinical settings.

## 2 Materials and methods

A qualitative study involving semi-structured interviews was conducted. The findings were systematically reported in alignment with the Consolidated Criteria for Reporting Qualitative Research (COREQ) checklist (Tong et al., 2007) (Additional file 1).

### 2.1 Participants and recruitment

Family medicine specialists and trainees were invited to participate in interviews from May 2023 to January 2024. Trainees are physicians currently undergoing specialized training to become family medicine specialists. Hereafter, we will refer to both family medicine specialists and trainees as GPs. Opportunistic sampling was used, aiming to include doctors based on various factors such as their years of experience, gender, and work environment. This approach combined elements of both purposive and convenience sampling, as it targeted specific criteria while leveraging available opportunities for inclusion. The study continued to enroll participants until data saturation was reached. Ten primary care facilities and 20 individual GPs (19 specialists and 1 trainee) were contacted via email. Facilities were encouraged to invite both specialists and trainees, though the exact number of participants reached is unknown. Three GPs declined due to time constraints, while the others did not respond. All agreed participants completed the study, and all interview data were included in the analysis.

### 2.2 Data collection

An interview guide featuring open-ended questions, derived from prior research findings (Bužančić and Ortner Hadžiabdić, 2022; Shrestha et al., 2022; Tangiisuran et al., 2022), was developed and pilot-tested with two GPs. While no new questions were introduced, some were reworded for clarity.

Physicians received invitations to participate in interviews via email, along with the interview guide and consent forms to sign in advance if they chose to participate. The interviews were conducted in Slovenian language by NJ (female) and lasted from 15 to 40 min. At the time of the study, NJ was employed as an Assistant and PhD student at the Faculty of Pharmacy, University of Ljubljana and had 3 years of research experience in pharmacy and qualitative research methods. She had no prior relationships with participants, except for one who was her former PhD professor. Efforts were made to maintain objectivity throughout the research process. Prior to the interviews, participants were briefed on NJ’s academic background and research interests to provide context for her role in the study. Interviews were conducted live in physicians’ clinics or at the Faculty of Pharmacy, or exceptionally online. No one besides the participant and the researcher was present during the interview. For qualitative analysis purposes, the interviews were audio-recorded, and no field notes were made. The questions focused on exploring GPs’ observations based on their clinical experience within their own practices. Although low-dose PPIs are available over the counter in Slovenia, the questions specifically addressed prescription-based PPI use.

### 2.3 Ethical considerations

The study was approved by the National Medical Ethics Committee of the Republic of Slovenia (Protocol number 0120-260/2023/6). Signed consents were obtained from all participants.

### 2.4 Data analyses

Verbatim transcripts were made in Microsoft Word and analysed with NVivo 11/12 Pro and were not shared with participants for their comments or corrections. Thematic analysis was conducted, with themes and subthemes created and coded into categories by NJ based on transcript information, which were later reviewed by NH for appropriateness. The analysis was inductive, using open coding principles to code the text, followed by refining the codes, removing duplicates, and consolidating them into higher hierarchical units, ultimately forming categories. Participants did not provide feedback on the findings. To maintain anonymity, the names of individuals and health institutions were not disclosed in the coded records. ChatGPT 4.0 by OpenAI was used solely for text formatting, grammar checks, and improving English clarity, with no involvement in content generation or data analysis.

## 3 Results

Nine interviews were conducted with Family medicine specialists (N = 6) or trainees (N = 3). The participant characteristics are detailed in Table 1. Four main categories were identified through thematic analysis: 1) Inappropriate prescribing of PPIs (number of references 30), 2) Facilitators for deprescribing PPIs (number of references 15), 3) Barriers to deprescribing PPIs (number of references 63), and 4) Feasibility of deprescribing PPIs (number of references 79). Categories with all themes and subthemes are listed in Supplementary Table 1 and further presented in the text (Additional file 2).

**TABLE 1:** Characteristics of participants.

Physicians (N = 9)	
Age in years (mean, SD, range)	45 (16.0) [27–72]
Gender (n, %) Female Male	6 (67) 3 (33)
Practitioner level (n, %) Family medicine specialists Trainees	6 (67) 3 (33)
Years practicing (mean, SD, range)	19 (15.7) [3–44]
Practice location^a^ (n, %) Urban Rural	6 (67) 3 (33)
Collaboration with clinical pharmacist (n, %) Yes No	7 (78) 2 (22)

SD: standard deviation.

^a^ Urban areas were defined as cities and suburbs based on the geographic setting of the GP, practices, while rural areas were defined as villages, small towns, and dispersed settlements.

### 3.1 Category 1: inappropriate prescribing of PPIs

GPs highlighted issues with PPI prescriptions, including excessive use, often in high doses, lack of deprescribing plans, and unverified medical indications. They also noted occasional low dosing, concurrent prescriptions of two PPIs, and unchecked drug interactions with PPIs.

#### 3.1.1 Theme 1: excessive prescribing (no. of references = 10)

GPs have noted the rapid and frequent routine prescription of PPIs among patients with polypharmacotherapy or those taking medicines, such as anticoagulants and non-steroidal anti-inflammatory drugs (NSAIDs), often questioning the necessity and dosage. They also highlighted that prolonged use contributes to widespread overuse, despite a few individuals needing long-term use.

“To have an indication to regularly take PPIs for many years is very rare, much rarer than our prescribing practices would suggest.” (GP 1, female, 49 years old).

#### 3.1.2 Theme 2:lack of deprescribing (no. of references = 7)

PPIs are typically provided without cessation plans, citing the example of their prescription during hospital stays as a short-term measure. However, their use often continues after discharge due to a lack of communication between patients and healthcare providers, and the absence of a systematic deprescribing process in clinical practice, as mentioned by GPs.

“Yes, (I observe irregularities in prescribing PPIs), such as starting patients on them (PPIs) without any plan to eventually discontinue them.” (GP 7, female, 55 years old).

#### 3.1.3 Theme 3: prescribed doses are too high (no. of references = 6)

GPs observed that PPIs are often not prescribed at the lowest effective dose in the prevention of gastrointestinal ulcers, particularly in patients with a low risk of gastrointestinal bleeding who are prescribed NSAIDs or antiplatelet therapy.

#### 3.1.4 Theme 4: not verifying the medical indication (no. of references = 4)

GPs acknowledged that in their routine practice, they encounter patients with prescribed PPIs that lack a valid indication, or they sometimes renew PPI prescriptions without verifying that an evidence-based indication is clearly documented in the medical records, which can lead to patients taking PPIs for several years.

#### 3.1.5 Theme 5: prescribed doses are too low (no. of references = 1)

One GP stated that PPI doses are sometimes prescribed too low for patients with a high risk of gastrointestinal bleeding, but this occurs less commonly than prescribing too high doses.

#### 3.1.6 Theme 6: concurrent prescribing of two PPIs (no. of references = 1)

One GP stated that she had previously overlooked that a patient was already taking another PPI when prescribing esomeprazole for *Helicobacter pylori* eradication, leading to the concurrent prescribing of two PPIs.

#### 3.1.7 Theme 7: not checking for drug interactions (no. of references = 1)

One GP mentioned that interactions between PPIs and other medicines are occasionally not checked in his clinical practice, despite their rarity.

### 3.2 Category 2: facilitators for deprescribing PPIs

**FIGURE 1:**
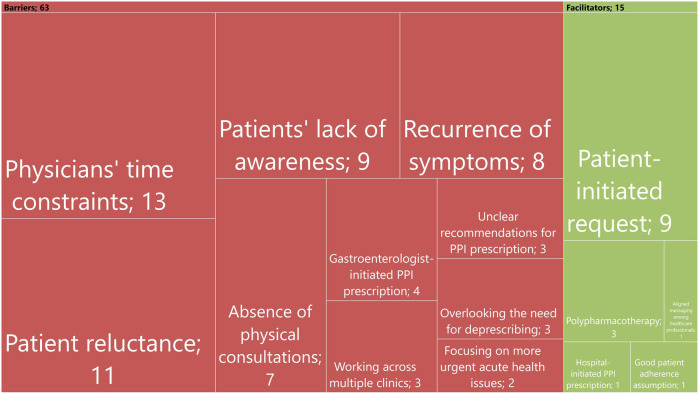
Summary of themes identified for barriers and facilitators of deprescribing. Each rectangle’s size reflects how often each theme was mentioned, with the numbers inside indicating the actual number of references.

GPs identified key facilitators for deprescribing PPIs, including patient-initiated request, polypharmacotherapy, aligned messaging among healthcare professionals, hospital-initiated PPI prescriptions, and expectations of good patient adherence (Figure 1).

#### 3.2.1 Theme 1: patient-initiated request (no. of references = 9)

Patient desires and requests to discontinue PPIs motivate GPs to intervene, initiate a conversation with the patient, review therapy, and tailor treatment to individual needs while considering patient preferences.

“If the patient requests it (deprescribing), we delve deeper and explore the background to assess if the medicine is truly necessary, and we’re also more motivated to deprescribe if there are reasons to do so.” (GP 8, male, 27 years old).

#### 3.2.2 Theme 2: polypharmacotherapy (no. of references = 3)

Polypharmacotherapy prompts GPs to identify deprescribing candidates, often including PPIs.

“One of the main reasons we pay attention and start thinking about deprescribing is when a patient is taking a lot of medicines … ” (GP 8, male, 27 years old).

#### 3.2.3 Theme 3: aligned messaging among healthcare professionals (no. of references = 1)

One GP noted that aligned messaging among healthcare professionals facilitates deprescribing because consistent communication from multiple sources increases patient trust and adherence to the deprescribing process.

“It’s beneficial if patients hear consistent information from different sources. When all of us—GPs, clinical specialists, and/or pharmacists—convey the same message, the likelihood of successful outcomes increases.” (GP 1, female, 49 years old).

#### 3.2.4 Theme 4: hospital-initiated PPI prescription (no. of references = 1)

Hospital-initiated PPI prescriptions prompt GPs to reassess the necessity of the medicine, particularly because they recognize that many patients receive them in the hospital but may not require them after discharge, as mentioned by one GP.

#### 3.2.5 Theme 5: good patient adherence expectation (no. of references = 1)

One GP suggests deprescribing PPIs if he assumes good patient adherence to the deprescribing scheme. According to his judgment, if the patient is competent and cooperative, he would consider deprescribing PPIs more often.

### 3.3 Category 3: barriers to deprescribing PPIs

GPs identified barriers to deprescribing PPIs, including time constraints, patient reluctance, lack of patient awareness, fear of symptom recurrence, absence of physical consultations when prescribing PPI, gastroenterologist-initiated PPI prescriptions, working across multiple clinics, overlooking the need for deprescribing, unclear recommendations, and prioritizing acute health issues (Figure 1).

#### 3.3.1 Theme 1: physicians’ time constraints (no. of references = 13)

The most common barrier mentioned by GPs was time constraints. Due to these constraints and to manage their patient load effectively, GPs may frequently renew PPIs prescriptions without a thorough review of the patient’s current medication needs. The deprescribing process, which requires time for detailed discussions with patients, often cannot be prioritized.

“The reality is that there is not always time for it - there should be, yes … But sometimes, there’s a rush, so much work awaits, there's a more urgent intervention, and you just say: “Okay, let’s postpone it for three months.” (GP 7, female, 55 years old).

#### 3.3.2 Theme 2: patient reluctance (no. of references = 11)

GPs face challenges when patients exhibit reluctance to have PPI deprescribed, often due to perceived reliance, necessity, or habit, and sometimes resort to continue prescriptions to avoid conflict.

“If someone is very dependent on PPI, it becomes challenging to deprescribe it, as they feel they cannot live without it.” (GP 1, female, 49 years old).

#### 3.3.3 Theme 3: patients’ lack of awareness (no. of references = 9)

Patients often embrace PPIs readily, considering them beneficial even if they do not fully understand their purpose, according to GPs. They commonly view them as protective agents alongside their regular therapy. Their limited awareness of the adverse effects of PPIs and the reason for their use leads them to not question the necessity for PPI use.

“I know many patients have told me: “I think I take this (PPI) because I'm on blood pressure medicine, and I take many medicines in general. So, because I take so many medicines, I also take this protection for my stomach,” and this protection sounds really good.” (GP 6, female, 40 years old).

#### 3.3.4 Theme 4: recurrence of symptoms (no. of references = 8)

GPs observe that patients fear symptom recurrence after stopping PPIs, particularly due to past experiences with rebound effects. This fear greatly affects patients’ willingness to engage in deprescribing. While GPs acknowledge this as a barrier for patients, they did not mention it as a barrier for themselves.

“Some patients find the rebound effect unbearable for the week or two it lasts. They constantly ask and after they’ve requested it ten times, you eventually let them continue with the PPI.” (GP 3, male, 33 years old).

#### 3.3.5 Theme 5: absence of physical consultations (no. of references = 7)

Patients often request PPIs online along with other regular therapy through digital healthcare services, such as electronic prescription platforms from specific primary care facilities, or by emailing their GP directly. This process allows them to request repeat prescriptions without face-to-face consultations. This limits the evaluation of ongoing therapy, potentially resulting in the prescription of unnecessary medicines, as indicated by GPs.

“Previously, when they came for written prescriptions, you still had some personal contact, and maybe you thought about it more when you were writing it – Do they really need the prescription? Now, you just click … ” (GP 8, male, 27 years old).

#### 3.3.6 Theme 6: gastroenterologist-initiated PPI prescription (no. of references = 4)

Gastroenterologist-initiated PPI prescriptions may cause GPs to hesitate to deprescribe PPI without specialist input because of their perceived authority and expertise.

“I am reluctant to discontinue what another specialist has introduced without consulting them. If prescribed by a gastroenterologist, I am less likely to deprescribe that PPI … ” (GP 4, female, 28 years old).

#### 3.3.7 Theme 7: working across multiple clinics (no. of references = 3)

GPs are hesitant to deprescribe when covering shifts at other clinics, especially due to their limited familiarity with patient histories. This, coupled with time constraints managing both their own clinic and that of their colleague’s, often leads them to prefer providing prescription refills and awaiting the return of the patient’s regular physician rather than making changes if they’re not deemed necessary. They acknowledge that this approach can lead to unnecessary PPI prescriptions.

#### 3.3.8 Theme 8: overlooking the need for deprescribing (no. of references = 3)

GPs mentioned they might overlook deprescribing PPIs because it is not their primary focus during therapy reviews. For instance, if a patient started PPI along with NSAIDs, the NSAIDs might be discontinued, but the PPI could remain unnoticed and become part of chronic therapy.

#### 3.3.9 Theme 9: unclear recommendations for PPI prescription (no. of references = 3)

GPs reported a lack of guidance on the necessity and dosage of PPIs alongside medicines such as oral corticosteroids, antiplatelets, or anticoagulants, or in patients with a history of gastrointestinal bleeding several years ago. This uncertainty could hinder the deprescribing process.

#### 3.3.10 Theme 10: focusing on more urgent acute health issues (no. of references = 2)

Patients often present to clinics with acute health concerns, which are not conducive to deprescribing PPIs. Without systematic protocols and exclusive follow-up appointments focused on deprescribing, GPs stated that this process can easily be overlooked.

### 3.4 Category 4: feasibility of deprescribing PPIs

**FIGURE 2:**
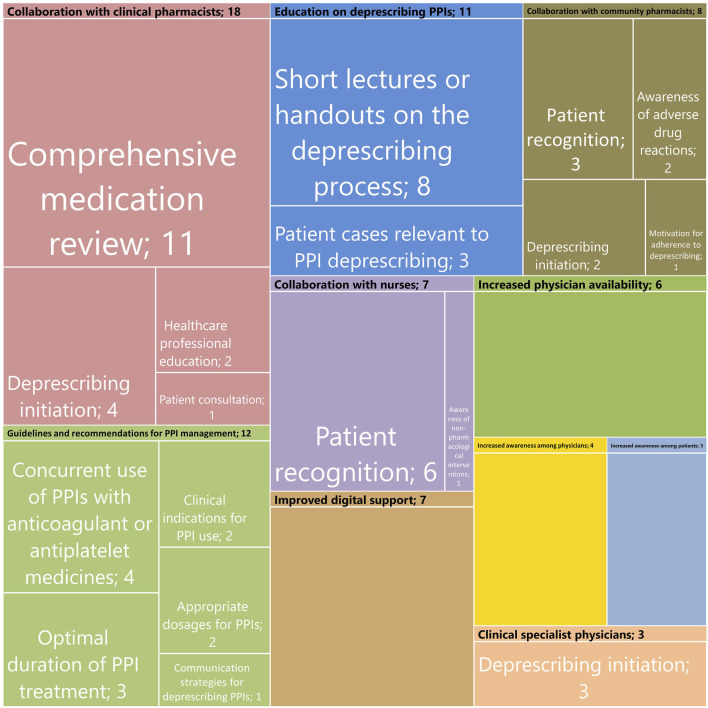
Summary of themes and subthemes identified for the feasibility of deprescribing PPIs. Each rectangle’s size reflects how often each theme and subtheme was mentioned, with the numbers inside indicating the actual number of references.

GPs identified several factors that could enhance the feasibility of deprescribing PPIs, including collaboration with clinical and community pharmacists, clear guidelines, educational resources, and support from nurses (Figure 2). Additionally, they believe educating both physicians and patients about the benefits of deprescribing, improved digital support, increased physician availability, and collaboration with other specialists in the deprescribing process would also make this process more effective (Figure 2).

#### 3.4.1 Theme 1: collaboration with clinical pharmacists (no. of references = 18)

GPs emphasize the pivotal role of collaboration with clinical pharmacists in enhancing the feasibility of deprescribing PPIs. They underscored pharmacists’ involvement in conducting comprehensive medication reviews, involving patient consultation, and providing deprescribing recommendations. Some suggest that clinical pharmacists could initiate deprescribing based on established protocols and educate healthcare professionals, further promoting deprescribing.

“We refer patients to medication review, during which clinical pharmacists evaluate and recommend dosage reductions, discontinuations, substitutions, and more. This is undoubtedly one of the most significant factors and the greatest help we can receive.” (GP 8, male, 27 years old).

#### 3.4.2 Theme 2: guidelines and recommendations for PPI management (no. of references = 12)

GPs stress the importance of evidence-based recommendations, particularly regarding concurrent PPI use with anticoagulants or antiplatelets. They seek clarity on the appropriate indication and treatment duration for PPI use, especially for indications dating back several years, and when chronic PPI use is genuinely necessary. They desire specific recommendations on dosage adjustments and administration frequency during the day. Some GPs also seek effective communication strategies to initiate PPI deprescribing discussions with patients.

“It would be ideal in the guidelines to specify - there’s still a dilemma about when to use 40 mg or 20 mg, whether to take 40 mg twice a day or 20 mg twice a day, and so on. Thus, the dose, frequency, and duration of PPI use.” (GP 8, male, 27 years old).

#### 3.4.3 Theme 3: education on deprescribing PPIs (no. of references = 11)

GPs feel overwhelmed by the amount of educational material they receive and prefer shorter, more practical resources. They believe that short lectures or handouts can effectively convey essential information, empowering them to confidently navigate deprescribing PPIs. Additionally, patient cases provide real-world context, aiding in understanding appropriate PPI deprescribing strategies.

#### 3.4.4 Theme 4: collaboration with community pharmacists (no. of references = 8)

GPs see the main role of community pharmacists in recognizing inappropriate PPI prescriptions and in guiding patients to consult their physicians about deprescribing. Community pharmacists should raise awareness of potential adverse drug reactions, ensuring that patients understand the risks of prolonged PPI use. Following established protocols, community pharmacists can initiate deprescribing discussions, as mentioned by a few GPs, and encourage patients to adhere to the deprescribing plan.

#### 3.4.5 Theme 5: collaboration with nurses (no. of references = 7)

Nurses, particularly those in model practices, could identify inappropriate PPI prescriptions as suggested by GPs. In Slovenia, model practices are expanded family medicine clinics where certified nurses help manage chronic conditions and conduct preventive exams. Additionally, raising awareness of non-pharmacological interventions for conditions like gastroesophageal reflux disease, where patient knowledge is often limited, could be very beneficial and contribute to a reduced need for PPIs.

“One possibility could involve asking a question or two on this topic, after which she (nurse) would inform me, and I would deprescribe PPI if there is no longer an indication.” (GP 5, male, 35 years old).

#### 3.4.6 Theme 6: improved digital support (no. of references = 7)

Enhanced digital support could streamline deprescribing PPIs by automatically flagging prolonged prescriptions, highlighting interactions, and providing immediate access to prescription history and any changes made to the medicine, according to GPs.

“There needs to be a mechanism that tells you when a medicine was introduced, how long it has been used, and how the prescriptions follow one another.” (GP 3, male, 33 years old).

#### 3.4.7 Theme 7: increased physician availability (no. of references = 6)

GPs believe that longer consultation times would enable more thorough medication reviews, thereby facilitating systematic deprescribing and strengthening patient trust for more successful outcomes.

“If I had more time with people, a better relationship would be built, more trust … We could then delve deeper into individual’s situation and health condition, and then it's also easier to say (whether to deprescribe).” (GP 7, female, 55 years old).

#### 3.4.8 Theme 8: increased awareness among physicians (no. of references = 4)

According to GPs, deprescribing is not a priority during outpatient consultation. By shifting mindsets, integrating deprescribing into routine care through education and reminders, and emphasizing its importance in patient consultations, deprescribing can become as routine as prescribing.

“Decision to initiate deprescribing largely depends on my judgment. Someone would have to convince me that it’s important, and then I would act.” (GP 5, male, 35 years old).

#### 3.4.9 Theme 9: increased awareness among patients (no. of references = 3)

GPs believe that when patients understand the risks and reasons for long-term PPI use, they are more likely to initiate discussions on deprescribing. This awareness grows with consistent information from healthcare professionals, especially doctors and pharmacists, and public health campaigns emphasizing informed decision-making regarding PPI use, as mentioned by GPs.

#### 3.4.10 Theme 10: clinical specialist physicians (no. of references = 3)

GPs seek specialist collaboration in deprescribing. They would appreciate clinical specialists initiating deprescribing or including it in their recommendations, especially if they had started the patient’s PPI therapy.

## 4 Discussion

In our study, GPs noted excessive and often inappropriate PPI prescribing, with a lack of deprescribing efforts. The primary facilitator for deprescribing PPIs was patient-initiated request to reduce medicines. Key barriers included GPs’ time constraints, patient reluctance, and a lack of patient awareness. To improve the feasibility of deprescribing PPIs, GPs have suggested collaborations within healthcare professionals and clearer guidelines. Additionally, educating both physicians and patients about the benefits of deprescribing, improved digital support, and increased physician availability are crucial to enhancing the feasibility of deprescribing PPIs, according to GPs.

### 4.1 Inappropriate prescribing of PPIs

GPs acknowledged the frequent and unnecessary prescription of PPIs, often at excessively high doses and for prolonged periods, and noted a significant lack of effort to deprescribe these medicines. Our findings align with broader concerns over PPI overuse, which also emphasize extensive use in high-risk groups such as the elderly (Hamzat et al., 2012) and cancer patients (Raoul et al., 2021), who are more prone to long-term use-related adverse events (Hayes et al., 2019; Hopkins et al., 2022; Chalabi et al., 2020). Despite the recognition of this issue and numerous calls for deprescribing PPIs (Farrell et al., 2017; Lee and McDonald, 2020; Targownik et al., 2022; Raoul et al., 2021), there is a critical gap between clinician awareness and implementation, as observed by the GPs in our study. Gendre et al. (2022) study using French national health data revealed no significant progress in deprescribing chronic PPI treatments over the past few years, underscoring the stagnation in addressing this issue. Therefore, there is a need to recognize strategies to enhance the feasibility of PPI deprescribing interventions and effectively implement existing recommendations (Farrell et al., 2017; Targownik et al., 2022).

### 4.2 Facilitators for deprescribing PPIs

The main facilitator for deprescribing PPIs, recognized by GPs in our study, was patient-initiated requests to reduce the number of medicines, with aligned messaging among healthcare professionals further facilitating the process. GPs can struggle with patient disagreements to deprescribing, making the decision-making process challenging (Fried et al., 2011); thus, when patients proactively request deprescribing and are suitable candidates, the negotiation phase is eliminated by aligning the goals of care with patient preferences. Patient-initiated requests indicate high patient engagement and reflect their values, which GPs should consider when evaluating deprescribing (Farrell et al., 2017). These requests can overcome GPs’ inertia and prompt immediate action from them to evaluate reducing the medication burden, which might otherwise be delayed due to other priorities (Anderson et al., 2014). Therefore, healthcare professionals should be aware that encouraging patients to actively participate in their care by discussing their medication burden, along with aligned messaging among all providers, will most effectively empower patients to initiate deprescribing PPIs when needed (Linsky et al., 2022).

### 4.3 Barriers to deprescribing PPIs

In our study, GPs identified important patient-centered barriers to deprescribing PPIs, including patient reluctance to deprescribing, lack of patient awareness, and fear of symptom recurrence, which are among the most significant obstacles to successful deprescribing reported in the literature (Tangiisuran et al., 2022). Weir et al. (2023) found that over 40% of older adults expressed doubts about deprescribing and valued their medicines highly. Patient reluctance often stems from the belief that medicine is beneficial or necessary for their condition (Reeve et al., 2013), especially when patients find PPIs more effective than other medicines they have tried (Boath and Blenkinsopp, 1997). Interestingly, Weir et al. (2023) noted that even patients who initially disagree with deprescribing recommendations still desire more communication, emphasizing the importance of engaging with hesitant patients. Specifically, discussing the rationale for deprescribing PPIs and guidance on managing symptom recurrence are the primary expectations from patients when discussing deprescribing PPIs (Thompson et al., 2020). Thus, increasing patient awareness of the risks associated with long-term PPI use and the benefits of deprescribing, along with providing a clear tapering plan, might help foster patient uptake (Farrell et al., 2017).

Physician- and system-centered barriers to deprescribing PPIs were also frequently cited by GPs in our study, with time constraints and the absence of physical consultations being the most significant. GPs face numerous challenges during their brief consultation times and heavy workloads (Raghunath et al., 2005; Anderson et al., 2014), especially when managing patients with multimorbidities and complex medication regimens (Doherty et al., 2020), making it difficult to prioritize deprescribing. Recognizing the burnout and overload GPs face, it is crucial to support them with strategies to assure appropriate initial PPI prescriptions, clear documentation of PPI indication, and its scheduled face-to-face reassessments, as inappropriate long-term PPI use often stems from the lack of initial deprescribing plans and the failure to reassess the necessity of PPIs (Lee and McDonald, 2020).

### 4.4 Feasibility of deprescribing PPIs

In our study, GPs were very engaged in finding ways to enhance deprescribing PPIs and overcome existing barriers. They highlighted collaboration with other healthcare professionals, particularly clinical pharmacists, as key. Most viewed pharmacists’ roles in conducting comprehensive medication reviews and providing deprescribing recommendations to physicians. Collaboration with clinical pharmacists has repeatedly proven to significantly enhance deprescribing feasibility (Bundeff and Zaiken, 2013; Hughes et al., 2011) by identifying inappropriate PPI prescriptions, actively participating in deprescribing and monitoring processes, and assisting in implementing best-practice recommendations in clinical practice (Farrell et al., 2017; Targownik et al., 2022). Community-based pharmacists and nurses can also contribute to feasibility of deprescribing PPIs by educating patients on non-pharmacological treatments and managing rebound symptoms, provide monitoring, and follow-up (Targownik et al., 2022; Bužančić et al., 2022; Coyle et al., 2019). Both literature and GP perspectives suggest that implementing collaborative frameworks in primary clinical practice is essential to enhance the feasibility of deprescribing interventions.

The GPs in our study emphasized evidence-based guidelines for appropriate PPI use, especially in the prophylaxis of gastrointestinal ulcers, to identify when PPI indications are no longer appropriate. They favoured short, step-by-step protocol handouts and expressed readiness to use them in routine practice. Additionally, GPs emphasized the need for enhanced digital support, such as flagging prolonged PPI prescriptions, highlighting interactions, and providing immediate access to prescription history with clear PPI indications. Some recommendations for PPI gastroprotection in patients taking antithrombotics have already been compared, with guidance provided (Targownik et al., 2022). However, new evidence, especially in oncology, continues to emerge and must be followed (Raoul et al., 2023). Interactions between PPIs and anticancer medicines becoming increasingly recognized, necessitating additional guidance for cancer patients. For patients undergoing polypharmacotherapy, many protocols already exist, and some are integrated into deprescribing clinical decision support tools for primary care, such as G-MEDSS for medication reviews (Kouladjian et al., 2022) and MedStopper for ranking medicines most appropriate for deprescribing (MEDSTOPPER, 2023). Additionally, tools such as Arriba PPI can engage GPs in discussing the risks of long-term PPI use and initiate deprescribing of unnecessary PPI (Heisig et al., 2023). In hospital settings, MedSafer (McDonald et al., 2022) has shown improved feasibility of deprescribing by screening patient medical histories and suggesting deprescribing opportunities. Expanding and validating these tools in primary care could help GPs by providing clearer guidance and simplifying the process, but their effectiveness depends on robust digital systems offering comprehensive access to patient histories and medication data.

Moreover, the GPs in our study emphasized that raising awareness among healthcare professionals and patients could enhance the feasibility of deprescribing PPIs. Patient-centered interventions, such as nationwide educational campaigns and direct-to-patient materials, are known to increase deprescribing rates (Wilsdon et al., 2017; Linsky et al., 2022). For provider-centered interventions, it is crucial to convince clinicians of the importance of deprescribing and provide effective motivation to implement it (Wilsdon et al., 2017). Our study also highlighted that by shifting healthcare professionals’ mindsets, integrating deprescribing into routine care through education and reminders, and emphasizing its importance in patient consultations, deprescribing can become as routine practice.

### 4.5 Implications for clinical practice and research

Fostering collaboration between GPs, clinical pharmacists, and other healthcare professionals is essential for comprehensive medication reviews and effective deprescribing recommendations. Developing and promoting evidence-based guidelines for deprescribing PPIs and their concurrent use with interacting medicines, especially anticoagulants or antiplatelets, is crucial. These guidelines should be concise, step-by-step protocol handouts for practical application in primary care. Encouraging patient-initiated requests to reduce medicines can facilitate deprescribing, thus increasing patient awareness of the risks of long-term PPI use, and the benefits of deprescribing are vital. Addressing the time constraints and other barriers that GPs encounter, despite their systematic nature, is a necessity. Advocating for increased physician availability and improved digital support, including electronic clinical decision support systems, could enhance the feasibility of deprescribing PPIs. Provider-centered interventions should aim to highlight the importance of deprescribing PPIs to clinicians and effectively motivate them to act.

## 5 Limitations

Participants were volunteers, potentially introducing selection bias due to their specific interest in deprescribing PPIs, thus limiting generalizability. Despite achieving data saturation, the small sample size of only nine participants is a limitation. The reported observations by GPs were based on their clinical experience and were not validated through an external study. Nonetheless, this study provides in-depth insights into the real-world challenges faced by GPs and suggests strategies for larger-scale, context-specific interventions. Our future research will focus on patient perspectives to gain insights into their experiences and attitudes, enhancing our understanding and suggesting further improvements to the feasibility of deprescribing PPIs.

## 6 Conclusion

There is a notable gap between clinicians’ awareness of inappropriate PPI use and the implementation of existing recommendations for deprescribing PPIs in clinical practice. GPs face significant time constraints, which are a primary barrier to prioritizing deprescribing PPIs. When GPs attempt to initiate deprescribing, patients are often reluctant, fear symptom recurrence, or are unaware of the risks of long-term PPI use and the benefits of deprescribing. Thus, deprescribing PPIs is mainly facilitated by patient-initiated requests to reduce the number of medicines and is not systematically implemented in clinical practice. GPs emphasized the need for collaboration with healthcare professionals, particularly clinical pharmacists, in the PPI deprescribing process. They stressed the importance of evidence-based recommendations for deprescribing PPIs and their concurrent use with medicines that could interact with the PPIs. Improved digital support, increased physician availability, and increased awareness among healthcare professionals and patients are essential for enhancing the feasibility of deprescribing PPIs.

## Data Availability

Transcripts from interviews are not publicly available to maintain the confidentiality of participants. Requests to access the datasets should be directed to nusa.japelj@ffa.uni-lj.si.

## References

[B1] Anderson K. Stowasser D. Freeman C. Scott I. (2014). Prescriber barriers and enablers to minimising potentially inappropriate medications in adults: a systematic review and thematic synthesis. BMJ Open 4 (12), e006544. 10.1136/bmjopen-2014-006544 PMC4265124 25488097

[B2] Boath E. H. Blenkinsopp A. (1997). The rise and rise of proton pump inhibitor drugs: patients' perspectives. Soc. Sci. Med. 45 (10), 1571–1579. 10.1016/s0277-9536(97)00094-4 9351147

[B3] Bundeff A. W. Zaiken K. (2013). Impact of clinical pharmacists' recommendations on a proton pump inhibitor taper protocol in an ambulatory care practice. J. Manag. Care Pharm. 19 (4), 325–333. 10.18553/jmcp.2013.19.4.325 23627578 PMC10438075

[B4] Bužančić I. Kummer I. Držaić M. Ortner Hadžiabdić M. (2022). Community-based pharmacists' role in deprescribing: a systematic review. Br. J. Clin. Pharmacol. 88 (2), 452–463. 10.1111/bcp.14947 34155673

[B5] Bužančić I. Ortner Hadžiabdić M. (2022). Development and validation of comprehensive healthcare providers' opinions, preferences, and attitudes towards deprescribing (CHOPPED questionnaire). Pharm. (Basel) 10 (4), 76. 10.3390/pharmacy10040076 PMC9326567 35893715

[B6] Chalabi M. Cardona A. Nagarkar D. R. Dhawahir Scala A. Gandara D. R. Rittmeyer A. (2020). Efficacy of chemotherapy and atezolizumab in patients with non-small-cell lung cancer receiving antibiotics and proton pump inhibitors: pooled *post hoc* analyses of the OAK and POPLAR trials. Ann. Oncol. 31 (4), 525–531. 10.1016/j.annonc.2020.01.006 32115349

[B7] Coyle C. Symonds R. Allan J. Dawson S. Russell S. Smith A. (2019). Sustained proton pump inhibitor deprescribing among dyspeptic patients in general practice: a return to self-management through a programme of education and alginate rescue therapy. A prospective interventional study. BJGP Open 3 (3), bjgpopen19X101651. 10.3399/bjgpopen19X101651 PMC6970585 31581112

[B8] Doherty A. J. Boland P. Reed J. Clegg A. J. Stephani A. M. Williams N. H. (2020). Barriers and facilitators to deprescribing in primary care: a systematic review. BJGP Open 4 (3), bjgpopen20X101096. 10.3399/bjgpopen20X101096 PMC7465575 32723784

[B9] Doherty A. S. Shahid F. Moriarty F. Boland F. Clyne B. Dreischulte T. (2022). Prescribing cascades in community-dwelling adults: a systematic review. Pharmacol. Res. Perspect. 10 (5), e01008. 10.1002/prp2.1008 36123967 PMC9485823

[B10] Farrell B. Pottie K. Thompson W. Boghossian T. Pizzola L. Rashid F. J. (2017). Deprescribing proton pump inhibitors: evidence-based clinical practice guideline. Can. Fam. Physician 63 (5), 354–364. 28500192 PMC5429051

[B11] Fried T. R. Tinetti M. E. Iannone L. (2011). Primary care clinicians' experiences with treatment decision making for older persons with multiple conditions. Arch. Intern Med. 171 (1), 75–80. 10.1001/archinternmed.2010.318 20837819 PMC3021478

[B12] Gendre P. Mocquard J. Artarit P. Chaslerie A. Caillet P. Huon J. F. (2022). (De)Prescribing of proton pump inhibitors: what has changed in recent years? an observational regional study from the French health insurance database. BMC Prim. Care 23 (1), 341. 10.1186/s12875-022-01941-2 36582006 PMC9800230

[B13] Haastrup P. Paulsen M. S. Begtrup L. M. Hansen J. M. Jarbøl D. E. (2014). Strategies for discontinuation of proton pump inhibitors: a systematic review. Fam. Pract. 31 (6), 625–630. 10.1093/fampra/cmu050 25192903

[B14] Hamzat H. Sun H. Ford J. C. Macleod J. Soiza R. L. Mangoni A. A. (2012). Inappropriate prescribing of proton pump inhibitors in older patients: effects of an educational strategy. Drugs Aging 29 (8), 681–690. 10.1007/BF03262283 22775478

[B15] Hayes K. N. Nakhla N. R. Tadrous M. (2019). Further evidence to monitor long-term proton pump inhibitor use. JAMA Netw. Open 2 (11), e1916184. 10.1001/jamanetworkopen.2019.16184 31774516

[B16] Heidelbaugh J. J. Kim A. H. Chang R. Walker P. C. (2012). Overutilization of proton-pump inhibitors: what the clinician needs to know. Ther. Adv. Gastroenterol. 5 (4), 219–232. 10.1177/1756283X12437358 PMC3388523 22778788

[B17] Heisig J. Bücker B. Schmidt A. Heye A. L. Rieckert A. Löscher S. (2023). Efficacy of a computer-based discontinuation strategy to reduce PPI prescriptions: a multicenter cluster-randomized controlled trial. Sci. Rep. 13, 21633. 10.1038/s41598-023-48839-2 38062116 PMC10703926

[B18] Hopkins A. M. Kichenadasse G. McKinnon R. A. Abuhelwa A. Y. Logan J. M. Badaoui S. (2022). Efficacy of first-line atezolizumab combination therapy in patients with non-small cell lung cancer receiving proton pump inhibitors: *post hoc* analysis of IMpower150. Br. J. Cancer 126 (1), 42–47. 10.1038/s41416-021-01606-4 34711947 PMC8727569

[B19] Hughes G. J. Belgeri M. T. Perry H. M. (2011). The impact of pharmacist interventions on the inappropriate use of acid-suppression therapy. Consult Pharm. 26 (7), 485–490. 10.4140/TCP.n.2011.485 21729849

[B20] Kostnapfel T. Albreht T. (2024). Poraba zdravil, predpisanih na recept v Sloveniji v letu 2023. Ljubljana: Nacionalni inštitut za javno zdravje.

[B21] Kouladjian O. Donnell L. Reeve E. Hilmer S. N. (2022). Development, validation and evaluation of the goal-directed medication review electronic decision support system (G-MEDSS)©. Res. Soc. Adm. Pharm. 18 (7), 3174–3183. 10.1016/j.sapharm.2021.09.004 34583897

[B22] Lee T. C. McDonald E. G. (2020). Deprescribing proton pump inhibitors: overcoming resistance. JAMA Intern Med. 180 (4), 571–573. 10.1001/jamainternmed.2020.0040 32091554

[B23] Linsky A. M. Kressin N. R. Stolzmann K. Pendergast J. Rosen A. K. Bokhour B. G. (2022). Direct-to-consumer strategies to promote deprescribing in primary care: a pilot study. BMC Prim. Care 23 (1), 53. 10.1186/s12875-022-01655-5 35317734 PMC8939089

[B24] McDonald E. G. Wu P. E. Rashidi B. Wilson M. G. Bortolussi-Courval É. Atique A. (2022). The MedSafer study-electronic decision support for deprescribing in hospitalized older adults: a cluster randomized clinical trial. JAMA Intern Med. 182 (3), 265–273. 10.1001/jamainternmed.2021.7429 35040926 PMC8767487

[B25] MEDSTOPPER (2023). Available at: http://medstopper.com/ (Accessed April, 2023)

[B26] Muthiah M. D. Zheng H. Chew N. W. S. Xiao J. Lim L. G. Tan H. C. (2021). Outcomes of a multi-ethnic Asian population on combined treatment with clopidogrel and omeprazole in 12,440 patients. J. Thromb. Thrombolysis 52 (3), 925–933. 10.1007/s11239-021-02472-w 33959860

[B27] Raghunath A. S. Hungin A. P. Cornford C. S. Featherstone V. (2005). Use of proton pump inhibitors: an exploration of the attitudes, knowledge and perceptions of general practitioners. Digestion 72 (4), 212–218. 10.1159/000089727 16286734

[B28] Raoul J. L. Guérin-Charbonnel C. Edeline J. Simmet V. Gilabert M. Frenel J. S. (2021). Prevalence of proton pump inhibitor use among patients with cancer. JAMA Netw. Open 4 (6), e2113739. 10.1001/jamanetworkopen.2021.13739 34132796 PMC8209575

[B29] Raoul J. L. Moreau-Bachelard C. Gilabert M. Edeline J. Frénel J. S. (2023). Drug-drug interactions with proton pump inhibitors in cancer patients: an underrecognized cause of treatment failure. ESMO Open 8 (1), 100880. 10.1016/j.esmoop.2023.100880 36764092 PMC10024146

[B30] Reeve E. To J. Hendrix I. Shakib S. Roberts M. S. Wiese M. D. (2013). Patient barriers to and enablers of deprescribing: a systematic review. Drugs Aging 30 (10), 793–807. 10.1007/s40266-013-0106-8 23912674

[B31] Scott I. A. Hilmer S. N. Reeve E. Potter K. Le Couteur D. Rigby D. (2015). Reducing inappropriate polypharmacy: the process of deprescribing. JAMA Intern Med. 175 (5), 827–834. 10.1001/jamainternmed.2015.0324 25798731

[B32] Shrestha S. Poudel A. Reeve E. Linsky A. M. Steadman K. J. Nissen L. M. (2022). Development and validation of a tool to understand health care professionals' attitudes towards deprescribing (HATD) in older adults with limited life expectancy. Res. Soc. Adm. Pharm. 18 (9), 3596–3601. 10.1016/j.sapharm.2022.03.002 35296385

[B33] Tangiisuran B. Rajendran V. Sha'aban A. Daud N. A. A. Nawi S. N. M. (2022). Physicians' perceived barriers and enablers for deprescribing among older patients at public primary care clinics: a qualitative study. Int. J. Clin. Pharm. 44 (1), 201–213. 10.1007/s11096-021-01336-w 34642869

[B34] Targownik L. E. Fisher D. A. Saini S. D. (2022). AGA clinical practice update on de-prescribing of proton pump inhibitors: expert review. Gastroenterology 162 (4), 1334–1342. 10.1053/j.gastro.2021.12.247 35183361

[B35] Thompson W. Nissen M. Haastrup P. Le J. V. Lundby C. Nielsen J. B. (2020). Discussing proton pump inhibitor deprescribing: the views of Danish GPs and older patients. BMC Fam. Pract. 21 (1), 160. 10.1186/s12875-020-01227-5 32770959 PMC7415175

[B36] Tong A. Sainsbury P. Craig J. (2007). Consolidated criteria for reporting qualitative research (COREQ): a 32-item checklist for interviews and focus groups. Int. J. Qual. Health Care 19 (6), 349–357. 10.1093/intqhc/mzm042 17872937

[B37] Weersma R. K. Zhernakova A. Fu J. (2020). Interaction between drugs and the gut microbiome. Gut 69 (8), 1510–1519. 10.1136/gutjnl-2019-320204 32409589 PMC7398478

[B38] Weir K. R. Shang J. Choi J. Rana R. Vordenberg S. E. (2023). Factors important to older adults who disagree with a deprescribing recommendation. JAMA Netw. Open 6 (10), e2337281. 10.1001/jamanetworkopen.2023.37281 37819657 PMC10568363

[B39] Wilsdon T. D. Hendrix I. Thynne T. R. Mangoni A. A. (2017). Effectiveness of interventions to deprescribe inappropriate proton pump inhibitors in older adults. Drugs Aging 34 (4), 265–287. 10.1007/s40266-017-0442-1 28220380

[B40] Wolfe M. M. (2024). Proton pump inhibitors: overview of use and adverse effects in the treatment of acid related disorders. Available at: https://www.uptodate.com/contents/proton-pump-inhibitors-overview-of-use-and-adverse-effects-in-the-treatment-of-acid-related-disorders (Accessed May 13, 2024).

