# Supplementary material for: Improving the feasibility of deprescribing proton pump inhibitors: GPs’ insights on barriers, facilitators, and strategies
Source: Front Pharmacol. 2024 Sep 20;15:1468750. doi: 10.3389/fphar.2024.1468750 (PMC11449877; doi:10.3389/fphar.2024.1468750)
Supplement: Supplementary file 1 [file DataSheet2.pdf]

Supplementary Material

1 **Additional file Supplementary Table** Summary of categories, themes, and subthemes (No. of participants = 9)

Supplementary Table 1 Summary of categories, themes, and subthemes (No. of participants = 9)

Category (No. of references)	Themes (No. of references)	Subthemes (No. of references)
**Inappropriate prescribing of PPIs (30)**	• Excessive prescribing (10)<br>• Lack of deprescribing (7)<br>• Prescribed doses are too high (6)<br>• Not verifying the medical indication (4)<br>• Prescribed doses are too low (1)<br>• Concurrent prescribing of two PPIs (1)<br>• Not checking for drug interactions (1)	
**Facilitators for deprescribing PPIs (15)**	• Patient-initiated request (9)<br>• Polypharmacotherapy (3)<br>• Aligned messaging among healthcare professionals (1)<br>• Hospital-initiated PPI prescription (1)<br>• Good patient adherence expectation (1)	
**Barriers to deprescribing PPIs (63)**	• Physicians' time constraints (13)<br>• Patient reluctance (11)<br>• Patients' lack of awareness (9)<br>• Recurrence of symptoms (8)<br>• Absence of physical consultations (7)<br>• Gastroenterologist-initiated PPI prescription (4)<br>• Working across multiple clinics (3)<br>• Overlooking the need for deprescribing (3)<br>• Unclear recommendations for PPI prescription (3)<br>• Focusing on more urgent acute health issues (2)	
**Feasibility of deprescribing PPIs (79)**	• Collaboration with clinical pharmacists (18)	○ Comprehensive medication review (11)<br>○ Deprescribing initiation (4)<br>○ Healthcare professional education (2)<br>○ Patient consultation (1)
	• Guidelines and recommendations for PPI management (12)	○ Concurrent use of PPIs with anticoagulant or antiplatelet medicines (4)<br>○ Optimal duration of PPI treatment (3)<br>○ Clinical indications for PPI use (2)<br>○ Appropriate dosages for PPIs (2)<br>○ Communication strategies for deprescribing PPIs (1)
	• Education on deprescribing PPIs (11)	○ Short lectures or handouts on the deprescribing process (8)<br>○ Patient cases relevant to deprescribing PPIs (3)
	• Collaboration with community pharmacists (8)	○ Patient recognition (3)<br>○ Awareness of adverse drug reactions (2)<br>○ Deprescribing initiation (2)<br>○ Motivation for adherence to deprescribing (1)
	• Collaboration with nurses (7)	○ Patient recognition (6)<br>○ Awareness of non-pharmacological interventions (1)
	• Improved digital support (7)<br>• Increased physician availability (6)<br>• Increased awareness among physicians (4)<br>• Increased awareness among patients (3)<br>• Clinical specialist physicians (3)	○ Deprescribing initiation (3)

PPI: proton pump inhibitor
