# Supplementary material for: Improving the feasibility of deprescribing proton pump inhibitors: GPs’ insights on barriers, facilitators, and strategies
Source: Front Pharmacol. 2024 Sep 20;15:1468750. doi: 10.3389/fphar.2024.1468750 (PMC11449877; doi:10.3389/fphar.2024.1468750)
Supplement: Supplementary file 2 [file DataSheet3.pdf]

frontiers

Supplementary Material

Additional file 3 GP interview guide (in English and Slovenian)

a) English version

Interview guide

Date of conversation:

**TOPIC**	**PROBING QUESTIONS**	**SPECIFIC QUESTIONS**
General awareness	What irregularities in the prescription of proton pump inhibitors (PPIs) do you observe in clinical practice?<br><br>How would you describe the term »deprescribing«?	If the term is unfamiliar, explain that »deprescribing« refers to the process of reducing the dosage or discontinuing the medicine.
Facilitators and barriers	How often and when do you decide to deprescribe PPIs in patients?	How does your perspective change (if at all) when the patient is also prescribed medicines that may have clinically significant interactions with PPIs, such as antiplatelet medicines or cancer immunotherapy?<br><br>What role does knowing who prescribed the PPI play in your decision-making process?<br><br>How does the patient's willingness to deprescribe PPIs influence your decision-making process?
	What are the barriers to more frequent deprescribing of PPIs?	
Collaboration with healthcare professionals	How can healthcare professionals (e.g., pharmacists, clinical pharmacists, nurses) assist you in deprescribing PPIs?	In what ways could they assist with the deprescribing process, for example, in identifying patients, managing the deprescribing process, and monitoring patient progress?
Knowledge	Can you describe a typical patient case where you deprescribed PPIs and explain the process you followed?	
	What additional information should be included in guidelines or other tools to assist with deprescribing PPIs?	
	Would you like additional training on deprescribing PPIs, and what content should such training include?	
Additional opinions	If you are involved in deprescribing PPIs in routine clinical practice, how do you think the feasibility of this intervention can be improved?	

b) Slovenian version

Vodnik za intervju

Datum pogovora:

TEMA	VPRAŠANJA	PODVPRAŠANJA
Splošna ozaveščenost	Kakšne nepravilnosti pri predpisovanju zaviralcev protonske črpalke (ZPČ) opažate v klinični praksi?<br><br>Kako bi opisali sl. pojem »odpredpisovanje« (ang. deprescribing)?	Če pojma ne pozna, razložimo, da se »odpredpisovanje« nanaša na zmanjševanje intenzivnosti zdravljenja oz. ukinjanje zdravila.
Spodbude in ovire	Kako pogosto in kdaj se pri bolniku odločite za odpredpisovanje ZPČ?	Kako se vaš pogled razlikuje (če se), če ima bolnik poleg ZPČ predpisana zdravila, ki lahko z ZPČ vstopajo v klinično pomembne interakcije, npr. antiagregacijska zdravila, imunoterapija raka?<br><br>Kakšno vlogo pri odločitvi ima vedenje o tem, kdo je predpisal ZPČ?<br><br>Kakšno vlogo pri odločitvi ima naklonjenost bolnika k odpredpisovanju ZPČ?
	Kaj so ovire, da se ne odločite za bolj pogosto odpredpisovanje ZPČ?	
Sodelovanje z zdravstvenimi delavci	Na kakšen način bi vam lahko zdravstveni delavci (npr. lekarniški ali klinični farmacevt, diplomirana medicinska sestra) pomagali pri odpredpisovanju ZPČ?	Na kakšen način bi vam lahko pomagali v procesu odpredpisovanja pri npr. prepoznavi bolnikov, vodenju bolnikov in sledenju bolnikov?
Znanje	Lahko opišete tipičen primer bolnika, pri katerem ste odpredpisali ZPČ in na splošno poveste, kako ste postopali?	
	Katere informacije v smernicah ali drugih orodjih za pomoč pri odpredpisovanju ZPČ bi morale biti še vključene?	
	Bi si želeli dodatnih izobraževanj o odpredpisovanju ZPČ in kakšne vsebine bi morala takšna izobraževanja zajemati?	
Dodatna mnenja	Če se lotevate odpredpisovanja ZPČ v rutinski klinični praksi, na kakšen način menite, bi lahko izboljšali izvedljivost te intervencije?	
